# Evolution of multiple omics approaches to define pathophysiology of pediatric acute respiratory distress syndrome

**DOI:** 10.7554/eLife.77405

**Published:** 2022-08-01

**Authors:** Jane E Whitney, In-Hee Lee, Ji-Won Lee, Sek Won Kong

**Affiliations:** 1 Medical Critical Care, Pediatrics, Boston Children’s Hospital Boston United States; 2 Department of Pediatrics, Harvard Medical School Boston United States; 3 Computational Health and Informatics Program, Boston Children’s Hospital Boston United States; 4 Department of Pharmacology, Faculty and Graduate School of Dental Medicine, Hokkaido University Sapporo Japan; Ashoka University India; Cedars-Sinai Medical Centre United States

**Keywords:** acute respiratory distress syndrome, pediatrics, biomarker, single-cell profiling, genetics, genomics

## Abstract

Pediatric acute respiratory distress syndrome (PARDS), though both common and deadly in critically ill children, lacks targeted therapies. The development of effective pharmacotherapies has been limited, in part, by lack of clarity about the pathobiology of pediatric ARDS. Epithelial lung injury, vascular endothelial activation, and systemic immune activation are putative drivers of this complex disease process. Prior studies have used either hypothesis-driven (e.g., candidate genes and proteins, in vitro investigations) or unbiased (e.g., genome-wide association, transcriptomic, metabolomic) approaches to predict clinical outcomes and to define subphenotypes. Advances in multiple omics technologies, including genomics, transcriptomics, proteomics, and metabolomics, have permitted more comprehensive investigation of PARDS pathobiology. However, omics studies have been limited in children compared to adults, and analyses across multiple tissue types are lacking. Here, we synthesized existing literature on the molecular mechanism of PARDS, summarized our interrogation of publicly available genomic databases to determine the association of candidate genes with PARDS phenotypes across multiple tissues and cell types, and integrated recent studies that used single-cell RNA sequencing (scRNA-seq). We conclude that novel profiling methods such as scRNA-seq, which permits more comprehensive, unbiased evaluation of pathophysiological mechanisms across tissue and cell types, should be employed to investigate the molecular mechanisms of PRDS toward the goal of identifying targeted therapies.

## Introduction

Pediatric acute respiratory distress syndrome (PARDS) was first defined in children in 2015 as the acute onset of parenchymal lung disease on chest x-ray with severe hypoxemia not explained by cardiac disease (Pediatric Acute Lung Injury Consensus Conference Group, 2015). PARDS compared to ARDS affecting adults has lower incidence (2–13 Pediatric Acute Lung Injury Consensus Conference Group, 2015; Schouten et al., 2016; Hough, 2017 vs. 17.9–81.0 Rubenfeld et al., 2009 ; Rubenfeld et al., 2009; Li et al., 2011 per 100,000 person-years) and mortality (18%–27% Pediatric Acute Lung Injury Consensus Conference Group, 2015 vs. 27%–45% Rubenfeld et al., 2009; Li et al., 2011). Sepsis is the most common cause of ARDS affecting adults while pneumonia is the most common cause of PARDS (Khemani et al., 2019). Epidemiologic differences between pediatric and adult ARDS may reflect differences in lung function, immune response, and disease mechanism between children and adults.

The onset of PARDS follows a severe physiological insult, which can take a variety of forms including pneumonia, aspiration, inhalation, sepsis, pancreatitis, transfusion, or trauma. Prior studies have noted differences in risk factors and clinical outcomes for children with distinct PARDS phenotypes (e.g., direct vs. indirect lung injury), which may reflect differences in pathobiology (Yehya et al., 2018; Whitney et al., 2020a). The fact that a relatively small proportion of children, who are at risk for PARDS after exposure to a severe physiologic insult, develop PARDS may reflect underlying biological differences in genetically determined host susceptibility.

PARDS mortality has decreased slightly over time (Pediatric Acute Lung Injury Consensus Conference Group, 2015; Khemani et al., 2019), possibly due to improvements in delivery of supportive care. Targeted pharmacologic therapies are lacking, which may reflect a lack of knowledge about the pathobiology of this complex disease process. Prior studies have provided some insights into the biological processes driving ARDS development in those at risk, clinical outcomes in pediatric and adults with ARDS, and phenotypic heterogeneity. However, limitations in methodology and scope exist, and we continue to lack clarity about which molecular mechanisms affecting which tissues and cell types at what time produce this complex disease process.

We sought to summarize the existing literature and data on molecular investigations of PARDS, which has included both hypothesis-driven and unbiased investigations. Adult ARDS literature is included where it supports or refutes pediatric data, or addresses questions not yet investigated in PARDS. We review candidate gene, candidate protein, and in vitro investigations testing hypotheses about PARDS risk, outcomes, and subphenotypes. We review genome-wide association studies (GWAS), transcriptomic, and metabolomic discovery investigations also addressing PARDS risk, outcomes, and subphenotypes. Then, using the list of candidate genes that have been investigated, we appraise the results of our own survey of publicly available gene databases to determine the concordance between genotype and phenotype across multiple tissues and cell types. Finally, we discuss limitations of the methods to date and describe some of the ways in which single-cell RNA sequencing (scRNA-seq) may address prior limitations. The overarching goal of this work is to compile what is known about PARDS using unbiased genomic techniques in order to elucidate disease mechanism with the goal of identifying potential treatment targets.

## Summary of research to date

### Hypothesis-driven investigations

#### Candidate gene studies

**Figure 1.**
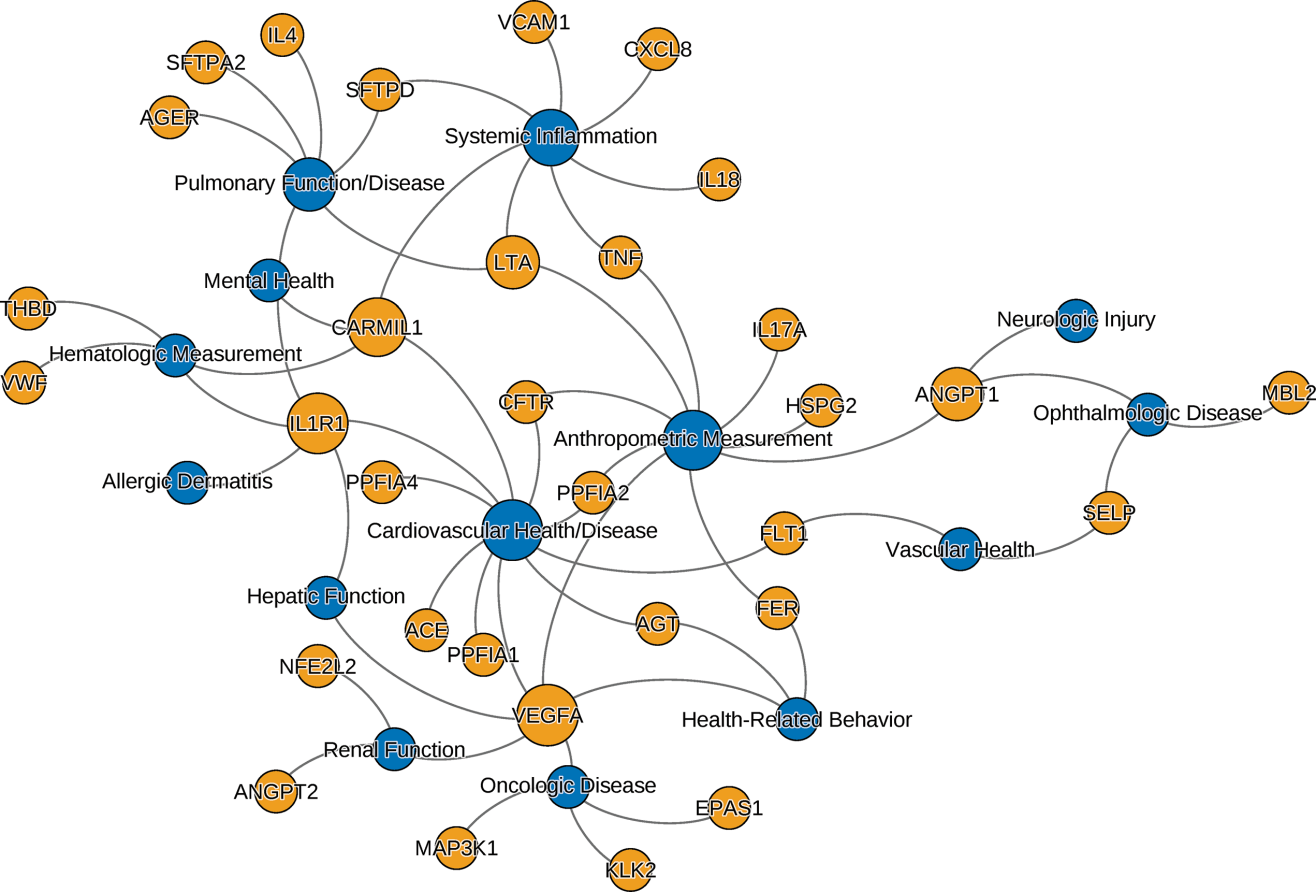
The relationships between ARDS candidate genes and phenotypes reported by genome-wide association studies. The nodes (circles) represent genes (light gray, gene symbols not italicized) or phenotypes (dark gray), and the associated genotype–phenotype pairs are connected by curved lines. For clear visualization, the original phenotypes reported by individual studies were simplified (Supplementary file 2). Node size is proportional to reported number of genotype–phenotype associations. The biological characteristics of ARDS, including pulmonary inflammation, systemic inflammation, and vascular endothelial activation, are associated with multiple candidate genes for ARDS. Abbreviations: ACE = angiotensin I-converting enzyme; AGER = advanced glycosylation end product-specific receptor; AGT = angiotensin; ANGPT = angiopoietin; ARDS = acute respiratory distress syndrome; CARMIL1 = capping protein regulator and myosin 1 linker 1; CFTR = cystic fibrosis transmembrane conductance regulator; CXCL8 = C-X-C motif chemokine ligand; EPAS1 = endothelial PAS domain protein 1; FER = feline encephalitis virus-related tyrosine kinase; FLT1 = fms-related receptor tyrosine kinase 1; HSPG = heparan sulfate proteoglycan; IL = interleukin; KLK2 = kallikrein-related peptidase 2; SFTP = surfactant protein; LTA = lymphotoxin alpha; MAP3K1 = mitogen-activated protein kinase 1; MBL = mannose binding lectin; NFE2L2 = nuclear factor, erythroid-like, BZIP transcription factor 2; PPFIA = protein tyrosine phosphatase receptor type F interacting protein alpha; SELP = selectin P; THBD = thrombomodulin; TNF = tumor necrosis factor; VCAM1 = vascular cell adhesion molecule 1; VEGFA = vascular endothelial growth factor A; vWF = von Willebrand Factor.

The development of PARDS in response to a severe physiologic insult is thought to reflect epithelial lung injury, systemic inflammation, and activation of the vascular endothelium. Candidate gene studies, therefore, have largely focused on genetic polymorphisms related to these processes, including surfactant protein function, pulmonary inflammation, systemic inflammation, and endothelial activation (Figure 1). While the requirement for a preceding physiologic insult precludes pedigree studies of PARDS, candidate gene studies (Table 1) have identified polymorphisms associated with the development of disease in some patients compared to others.

**Table 1.** Key Pediatric Studies.

Approach	Candidate Gene(s)	Sample Size	Findings (Adjusted OR and CI)	Author, Journal*, Year
HYPOTHESIS-DRIVEN				
Candidate Gene	*TNF, LTA*	490 intubated with sepsis, 610 healthy controls (total 1100)	Protective effect of *TNF-308GA* against ARDS in infants: OR 0.2, *P*=0.001	Azevedo et al., 2012
	*ACE*	60 with ARDS, 60 healthy controls (total 120, all <15 years)	I/D genotype not increased in ARDS: rate 0.4 (ARDS) vs. 0.3 (controls), *P*=NS	Cruces et al., 2012
	*SFTPB*	395 with pneumonia: 37 requiring mechanical ventilation, 26 with ALI/ARDS	Two linkage disequilibrium-tag SNPs associated with mechanical ventilation: GTGCGCG AOR = 2.62, CI 1–6.8, ATATAAG AOR = 3.1, CI 1–8.9	Dahmer et al., 2011
	*SFTP*	248 children <2 years with acute respiratory failure, 468 newborn healthy controls	34 interactions among 3 SNPs of *SFTPA1, SFTPA2, SFTPC* associated with acute respiratory failure (*P*=0.000000002–0.05) and pulmonary dysfunction after discharge (*P*=0.00002–0.03)	Gandhi et al., 2020
	*ACE*	13 Caucasian children with ARDS, 30 acute hypoxemic respiratory failure, 186 ICU controls (total 216)	I/D polymorphism not associated with acute hypoxemic respiratory failure or ARDS	Plunkett et al., 2008
Candidate Protein	*IL8*	480 with acute respiratory failure	Increased IL8 associated with mortality, duration mechanical ventilation, ICU LOS but not ARDS diagnosis	Flori et al., 2019
	many	3 cohorts: 46 with sepsis ARDS, 54 with sepsis without ARDS, and 19 ICU controls (total 119)	ANGPT2, ANGPT2/1 ratio higher in ARDS; ANGPT2, ANGPT2/1 ratio, VWF, ESM1 predicted complicated course in sepsis; in sepsis ARDS, FLT1 decreased more quickly and VWF, THBD decreased more slowly in those with complicated course	Whitney et al., 2020a
	*ANGPT1, ANGPT2, VCAM1, vWF*	2 cohorts of patients with ARDS: 52 direct, 46 indirect lung injury (total 98)	ARDS with indirect lung injury associated with increased ANGPT2/1 ratio, VCAM1, vWF (sensitivity 0.9, CI 0.8–1.0, specificity 0.8, CI 0.7–0.9)	Whitney et al., 2020a
	*AGER, ANGPT2*	82 with ARDS	Increased AGER, ANGPT2 associated with non-survival, organ failures in children with ARDS	Yehya et al., 2016
	*CCL3, HSPA1b, IL8*	153 with ARDS	Mortality associated with increased CCL3, HSPA1B, IL8, and older age in children with ARDS	Yehya et al., 2018
	many	235 with ARDS	Identified MMP profile associated with mortality (AOR 4.0, CI 2.1–7.6)	Zinter et al., 2019
	*ANGPT2, VEGF, VWF*	259 with ARDS, 25 status post HCST	Early ANGPT2 (OR 3.7, CI 1.1–11.5) and increasing ANGPT2 associated with mortality (AOR 3.3, CI 1.2–9.2), especially among HCST (AOR 16.3, CI 1.3–198)	Zinter et al., 2016
In Vitro Studies		Neutrophils and tracheal aspirates from 20 ARDS viral pneumonia with or without bacterial co-infection	In bacterial co-infection: (1) neutrophils more activated with impaired bacterial killing, respiratory burst, (2) aspirates with higher neutrophil elastase and myeloperoxidase, (3) neutrophils transmigrated into aspirate with decreased burst/killing of *H. influenzae, S. aureus*	Grunwell et al., 2019
		Tracheal aspirates from 42 intubated children with, 35 without ARDS (total 77)	Increased *STAT1* phosphorylation, markers of neutrophil degranulation and activation, NET release. Higher airway NETs associated with fever ventilator-free days	Grunwell et al., 2019
UNBIASED				
Gene Expression		28 intubated with, 26 without ARDS (total 54)	Using tracheal aspirates, a 62-gene signature to identify ARDS was developed to achieve cross-validation AUC 0.8, CI 0.6–0.9	Grunwell et al., 2021
		67 with sepsis and acute hypoxemic respiratory failure	Two identified endotypes differentially associated with mortality (OR 8, CI 1.6–41), complicated course (OR 4.2, 1.2–14.9)	Yehya et al., 2019
		96 with ARDS	Three identified sub-phentoypes associated with different clinical characteristics, outcomes	Yehya et al., 2020

Abbreviations: ACE = angiotensin-converting enzyme; AGER = advanced glycosylation end-product specific receptor; ALI = acute lung injury; ANGPT = angiopoietin; AOR = adjusted odds ratio; AUC = area under the receiver operator characteristic curve; ARDS = acute respiratory distress syndrome; CCL3 = C-C motif chemokine ligand 3; CI = 95% confidence interval; ESM1 = endothelial cell specific molecule 1; FLT1 = fms related receptor tyrosine kinase 1; HCST = hematopoietic stem cell transplant; HSPA1B = heat shock protein family A (Hsp70) member 1B; ICU = intensive care unit; IL = interleukin; LOS = length of stay; LTA = lymphotoxin alpha; MMP = matrix metalloproteinase; NET = neutrophil extracellular trap; OR = unadjusted odds ratio; SFTP = surfactant protein; SNP = single nucleotide polymorphism; SFTP = surfactant protein; THBD = thrombomodulin; TNF = tumor necrosis factor; TNFRSF1A = TNF receptor superfamily member 1A; VCAM1 = vascular cell adhesion molecule 1; VWF = von Willebrand Factor.

* Journal Titles are abbreviated according to U.S. National Library of Medicine convention.

Surfactant is a mixture of phospholipids and surfactant proteins (SP-A, SP-B, SP-C, and SP-D), and is critical for lowering surface tension at the alveolar–epithelial interface and protection against pathogens (Whitsett and Weaver, 2015). Patients with inherited surfactant deficiency demonstrate severe if not lethal respiratory failure. Surfactant lipids and proteins are produced by type II alveolar cells, and the surfactant lipid transporter (ABCA3) is required to form lamellar bodies that are secreted into the airways. Thus, variants in the genes encoding surfactant proteins A through D and ABCA3 have been of interest in ARDS. The *SFTPA1*, *SFTPA2*, *SFTPB*, *SFTPC*, and *SFTPD* genes encode SP-A1, SP-A2, SP-B, SP-C, and SP-D proteins, respectively. Among nearly 400 children with pneumonia, of whom the most severely affected had PARDS and need for mechanical ventilation, an association was demonstrated between *SFTPB* Single nucleotide polymorphisms (SNPs) and need for mechanical ventilation (Dahmer et al., 2011). In another study comparing children with acute respiratory failure and healthy newborn controls, SNPs in *SFTPA1*, *SFTPA2*, and *SFTPC* were associated with acute respiratory failure and pulmonary dysfunction after hospital discharge (Gandhi et al., 2020).

The angiotensin I-converting enzyme (ACE) degrades bradykinin and catalyzes angiotensin I–II, a potent vasoconstrictor with proinflammatory and profibrotic effects. High levels of circulating ACE are associated with pulmonary inflammation, and ACE is also highly expressed by endothelial cells composing the pulmonary microvasculature (Aird, 2007). The insertion/deletion (I/D) variant (rs4646994, a 287–289 basepair Alu-type repeat sequence in intron 16) in the *ACE1* gene has functional significance such that the D allele is associated with increased ACE activity and higher ACE levels in plasma and tissue. The association between D allele and ARDS risk and outcomes has been tested in several observational studies. In children, the I/D variant failed to demonstrate association with PARDS status but increased frequency of D allele was associated with PARDS severity (Cruces et al., 2012; Plunkett et al., 2008). An adult study confirmed the association between the DD genotype, increased risk for ARDS, and increased mortality from ARDS (Adamzik et al., 2007; Marshall et al., 2002) but did not test the I/D variant.

Candidate genes associated with systemic inflammation have been of long-standing interest in PARDS. Systemic inflammation often accompanies pulmonary inflammation, and systemic inflammatory processes such as sepsis are risk factors for PARDS. Tumor necrosis factor-α (TNF-α), encoded by the *TNFA* gene, is a pleiotropic cytokine and a primary mediator of systemic inflammation. Risk for PARDS in a cohort of intubated children with sepsis was associated with *TNFA*-863 C>A genotype (rs18000630), and the *TNFA*-308 G>A genotype (rs1800629) had a protective effect against mortality in sepsis-associated PARDS (Azevedo et al., 2012).

The activation of coagulation system with microthrombi causing tissue hypoxia contributes to pulmonary vascular injury in PARDS. The endothelial protein C receptor (EPCR, encoded by the *PROCR* gene) plays a cytoprotective role in sepsis by activating protein C (APC) and mediating APC effects. In the presence of thrombomodulin (TM encoded by the *THBD* gene), thrombin activates protein C to APC. We are not aware of studies investigating the association between candidate genes involved in coagulation and PARDS. Adult ARDS mortality has been associated with two *THBD* (rs1042580 and rs3716123) and one *PROCR* (rs9574) SNPs (Sapru et al., 2016).

A result of systemic inflammation, especially inflammation that occurs as a response to infection, is the activation of the vascular endothelium. Endothelial activation has been the focus of many candidate gene studies in adult but not yet pediatric ARDS, including those which have sought to determine its role in producing the disease in patients with risk factors. The *ANGPT2* gene encodes angiopoetin-2 (ANGPT2), which has been associated with loss of vascular integrity, increased permeability, and potentiation of vascular lung injury. In adults with sepsis, *ANGPT2* intronic SNPs (rs2442608 and rs2442630) have been associated with increased susceptibility to acute lung injury (ALI) (Meyer et al., 2011) or ARDS across multiple cohorts (Su et al., 2009), one in which plasma ANGPT2 was implicated as a causal intermediate (Reilly et al., 2018). In adults with trauma or sepsis as risk factors for ARDS, the development of ARDS was associated with *ANGPT2* (Tejera et al., 2012) and blood type A in White patients (Reilly et al., 2021), potentially reflecting vascular inflammatory changes. *FLT1* encodes soluble fms-like tyrosine kinase (sFLT, also known as vascular endothelial growth factor [VEGF] receptor), which competitively inhibits VEGF to promote vascular quiescence. An SNP in *FLT1* (rs9513106) was associated with reduced susceptibility to ARDS in a cohort study involving adults of Spanish and Western European descent with sepsis (Hernandez-Pacheco et al., 2018).

As a whole, candidate gene studies have demonstrated associations between the development of ARDS in patients with clinical risk factors and polymorphisms implicated in pulmonary inflammation, systemic inflammation, or endothelial activation. These associations have been demonstrated in multiple cohorts with varying risk factors for ARDS. However, the association of specific SNPs with ARDS has not been adequately replicated, and pediatric studies have been few. A strength of candidate gene studies is that they are highly feasible and useful when sample size is small. Notable disadvantages of candidate gene studies are false positives and poor replication. Furthermore, candidate gene studies are hypothesis-driven, investigating only genes of interest based on prior knowledge, and thus may unduly emphasize known biological themes while failing to address what has not yet been hypothesized.

#### Protein biomarker studies

Candidate protein studies of PARDS (Table 1) are numerous because they are highly feasible. Protein biomarkers are assayed from peripheral blood of patients with or at risk for PARDS, which is relatively noninvasive and low cost. Outcomes of interest for candidate protein studies have included: (1) whether PARDS develops in patients with clinical risk factors, (2) predictors of PARDS outcomes, and (3) distinguishing subphenotypes of PARDS to elucidate differences in pathobiology, clinical characteristics, and outcomes.

The development of ARDS in children and adults with clinical risk factors for the syndrome has been associated with candidate protein biomarkers reflecting endothelial activation, systemic inflammation, and epithelial lung injury. Limited number of studies and small sample sizes in pediatric ARDS reflects lower incidence of ARDS in children compared to adults as well as challenges associated with pediatric research (e.g., smaller circulating blood volume, less frequent blood draws).

Early increase in endothelial biomarkers ANGPT2 and ANGPT2/ANGPT1 ratio was associated with the development of PARDS in our prior study of children with extrapulmonary sepsis (Whitney et al., 2020b). Among those with PARDS, persistent organ dysfunction or death was associated with rapid decline in endothelial biomarker sFLT, and slow decline in von Willebrand Factor (vWF) and TM, which reflect the activation of the coagulation system (Whitney et al., 2020b). In a cohort of children, who underwent hematopoietic stem cell transplant, PARDS mortality was associated with both early elevation in and subsequent increase of ANGPT2 (Zinter et al., 2016). A profile of matrix metalloproteinases (MMPs) associated with multiple markers of inflammation and endothelial activation and predicted severe morbidity or mortality in a cohort of 235 children with PARDS (Zinter et al., 2019). One study of adults with ALI (Supplementary file 1) confirmed the association between increased ANGTP2 and mortality, modified by the presence of infection (Calfee et al., 2012).

Biomarkers of systemic inflammation have been studied in peripheral blood and bronchoalveolar lavage (BAL) fluid. In a cohort of children with acute respiratory failure, circulating interleukin-8 (IL-8) level was associated with worse outcomes but not PARDS status (Flori et al., 2019). The association of IL-8 with poor outcome from PARDS in the context of sepsis was confirmed in a larger cohort (Yehya and Wong, 2018).

A biomarker associated with pulmonary epithelial injury, receptor for advanced glycation end-products (RAGE), is encoded by the *AGER* gene and has activity in the lung. Yehya et al. found increased RAGE and ANGPT2 to be associated with nonsurvival and increased number of nonpulmonary organ failures in PARDS (Yehya et al., 2016). Three studies of septic adults (Supplementary file 1) supported these findings in demonstrating the association between increased RAGE and ARDS status (Uchida et al., 2006; Jones et al., 2020; Ware et al., 2013).

#### Defining endotypes with biomarkers

The concept of endophenotype was proposed as ‘measurable components unseen by the unaided eye along the pathway between disease and distal genotype (Gottesman and Gould, 2003)’ while an endotype is a subtype of disease defined by molecular pathobiology of treatment response (Anderson, 2008). Distinguishing endophenotypes has been of interest in ARDS, and endotypes of PARDS have been identified, which are associated with distinct clinical factors and outcomes (Yehya et al., 2018; Whitney et al., 2020a; Yehya et al., 2018) potentially reflecting differences in pathobiology (Table 1). First, Wong et al. used a large panel of biomarkers to define endotypes in pediatric sepsis, which were associated with differential mortality and response to therapies, including steroids (Wong et al., 2015; Wong et al., 2012). Yehya et al., 2019, recognizing sepsis as the most common cause of PARDS, used similar methods to define an endotype associated with poor outcomes from acute hypoxemic respiratory failure in septic children, identified by IL-8, C-C chemokine ligand 3 (CCL3), and heat shock protein 70 kDa 1B (HSPA1B), and age. The identifying characteristics were then tested in a PARDS cohort with good prediction of mortality (Yehya and Wong, 2018).

We previously reported that elevation in endothelial biomarkers ANGPT2/ANGPT1 ratio, VCAM1, and vWF distinguished children with ARDS due to indirect (e.g., extrapulmonary sepsis or trauma, shock, transfusion, pancreatitis) compared to direct (e.g., pneumonia, aspiration, drowning) lung injury (Whitney et al., 2020a). An adult ARDS study with complementary results (Supplementary file 1) showed lower ANGPT2 and higher SP-D in those with direct lung injury (Calfee et al., 2015). Biomarkers reflecting systemic inflammation and endothelial activation have been associated with worse clinical outcomes in adult ARDS (Supplementary file 1) with a differential response to therapies (Bos et al., 2017; Calfee et al., 2014; Famous et al., 2017; Sinha et al., 2020; Sinha et al., 2018).

In summary, protein biomarker studies in PARDS have been numerous and are highly feasible. They have described a pattern of epithelial and endothelial perturbations as well as systemic inflammation, which mirrors the findings of protein biomarker studied first conducted in adults with ARDS. Given known differences in ARDS epidemiology between children and adults and the putative influence exerted by the developing lung and immune systems, it is likely that biological differences between pediatric and adult ARDS exist. A precision medicine approach using unbiased omics methods may better define PARDS pathobiology for each patient as disease progresses or resolves.

#### In vitro studies

ARDS is typified by pulmonary neutrophilia, and in vitro studies (Table 1) have focused on clarifying the mechanisms of neutrophil activation, lack of neutrophil clearance by macrophages, and the role of neutrophil extracellular traps (NETs). NETs are webs of extracellular fibers composed of DNA with histones, myeloperoxidase (MPO) and neutrophil elastase (NE), which are important for immune response to infection but worsen inflammation when they persist (Brinkmann et al., 2004; Liu et al., 2016). Grunwell et al. examined neutrophils from children with PARDS secondary to viral lower respiratory tract infection with or without bacterial pneumonia, examining transmigration through their model airway with cell-free tracheal aspirate fluid (Grunwell et al., 2019). Patients with bacterial pneumonia had neutrophils with increased markers of activation and a defective respiratory burst, with airway fluid containing higher MPO and NE activity and decreased killing of *H. influenzae* and *S. aureus* (Grunwell et al., 2019). In a follow-up study comparing tracheal aspirates from intubated children with and without pediatric ARDS, increased type I interferon signaling (increased phosphorylation of *STAT1*), increased NET release (Grunwell et al., 2021). Increased markers of neutrophil activation and degranulation were observed in children with PARDS, also associated with fewer ventilator days in patients with higher airway NE (Grunwell et al., 2021). Similar findings were documented in lower airway neutrophils and neutrophils exposed to airway fluid from adults with vs. without ARDS (Supplementary file 1), including decreased apoptosis and macrophage activity and increased NET formation in ARDS (Grégoire et al., 2018).

These recent investigations represent an exciting development because they elucidate some of the pathobiological changes associated with lower respiratory tract infection that make a host vulnerable to ARDS. However, experiments were limited by study samples that were small and homogeneous, the inability to see changes over time, the possibility that cell signaling happens differently in vitro and in vivo, and a priori determination of the signaling pathways of interest. While this method is not easily applied to individual patients with PARDS, in vitro studies clarify aspects of disease mechanisms where observational studies cannot. Furthermore, in vitro studies can generate hypotheses to be followed-up in future clinical studies.

### Unbiased investigations

#### Genome-wide association studies

Several GWAS have been completed in adults but not children with ARDS (Supplementary file 1). In a multiphase study using GWAS to compare European-American adults with trauma-associated ALI to controls, ALI was associated with rs47191 in the *PPFIA1* gene, which encodes liprin-α-1, a protein involved in cell–matrix interactions and cell adhesion (Christie et al., 2012). A study of African American patients with ARDS compared to at-risk controls found ARDS to be associated with and a coding SNP (rs2228315) in the *SELPLG* gene, which regulates neutrophil adherence and diapedesis across the vascular endothelium (Bime et al., 2018). *SELPLG* knockout mice showed significantly reduced LPS-induced inflammatory lung injury compared with wild-type mice, and a neutralizing antibody against PSGL-1 (P-selectin glycoprotein ligand 1) reduced lung inflammation. In a comparison of European adults with ARDS and at-risk septic patients, an SNP in *FLT1* was associated with reduced susceptibility to ARDS (Guillen-Guio et al., 2020).

Together, these studies highlight a possible role for endothelial and pulmonary epithelial changes in genetic susceptibility to ARDS. Although GWAS permits an unbiased investigation of the genetic risks than candidate gene studies, several limitations make it difficult to implement for investigating ARDS and PARDS. First, sample sizes required for case and control groups are large relative to the prevalence of ARDS. Second, biological interpretation of risk alleles and understanding their relationship to associated genes in disease development is challenging. Third, it is important to understand the impact of genetic variants on various tissue types involved in a complex disease like ARDS which affects not only the lungs but peripheral vasculature as well.

#### Genome-wide gene expression studies

Analysis of microarray data from children with PARDS highlighted differential gene expression associated with biological processes implicated in disease pathogenesis and outcomes (Table 1). In evaluating a cohort of children with sepsis and acute hypoxemic respiratory failure, Yehya et al. identified two distinct transcriptomic profiles differentially associated with mortality and persistent organ dysfunction (Yehya et al., 2019). Subsequently, the same group evaluated a cohort of PARDS patients using peripheral blood transcriptome to identify three subgroups that were associated with distinct baseline clinical characteristics and ARDS outcomes (Yehya et al., 2020). The first subgroup was characterized by enriched adaptive immune response and persistent hypoxemia. The second subgroup was characterized by the activation of complement-related pathways, and the third exhibited suppression of adaptive immune and T-cell receptor pathways, which was associated with improved survival.

To discover transcriptomic signature in airway cells, Grunwell et al., 2021 collected tracheal aspirate samples from 28 patients with PARDS and 24 without PARDS. The top-ranked gene in the primary airway cells was *IL17A,* and a 62-gene prediction model for PARDS was developed, which was enriched with the genes in cytokine–cytokine receptor interaction. A neutrophil reporter assay was used to monitor gene expression changes seen in healthy donor neutrophils exposed to airway fluid patients with and without ARDS. The most significantly altered gene in the neutrophil reporter assay was *NT5E*. This preliminary study highlights a transcriptomic signature of PARDS in airway cells and neutrophils that could help identify future therapeutic targets.

Together, gene expression studies have strengthened existing hypotheses about mechanism of disease and outcomes in PARDS. Though some findings were tested in animal models, replication in humans is needed to validate the findings. Transcriptome analysis was performed on peripheral blood in most studies though Grunwell et al. examined airway epithelia. Future studies should continue evaluating differences across multiple tissue types to enhance understanding of ARDS pathogenesis in the human organism as a whole.

#### Metabolomics

High-resolution mass spectrometry can quantitatively measure hundreds to tens of thousands of small metabolites for diverse chemical species in tissue and liquid biopsy samples (Kong and Hernandez-Ferrer, 2020). To our knowledge metabolomics have not been used to study PARDS. Previous studies of adult ARDS (Supplementary file 1) used targeted metabolomics platforms with relatively limited chemical space coverage (Metwaly and Winston, 2020). Recently, Rogers et al. used an untargeted metabolomics platform to compare the profile of pulmonary edema fluid collected at the time of endotracheal intubation for 16 adults with ARDS compared to 13 with cardiogenic pulmonary edema. They found a high metabolite endotype characterized by higher concentration of lipids, amino acids, and carbohydrates to be associated with higher mortality from ARDS (Rogers et al., 2017).

A strength of metabolomic studies is that they are relatively unbiased and produce a large amount of data per patient. Current work is limited to a single fluid in a small cohort, which limits generalizability and replication may be difficult. Future precision medicine approaches to ARDS could include, but should not be limited to, metabolomic investigation of multiple tissue types in concert with genomic, transcriptomic, and protein biomarker analyses.

### Interrogation of genomic knowledge databases

Decades of clinical research have proven the utility of ARDS candidate genes to define endophenotypes, predict clinical outcomes, and discover treatment targets. These candidate genes are essential for normal lung function and immune responses, yet many genes have pleiotropic activities in diverse cell types, tissues, and organs.

We searched the NHGRI-EBI GWAS catalog (https://www.ebi.ac.uk/gwas, last accessed: June 8, 2021) to find genotype–phenotype (GP) associations for the candidate genes. Though candidate genes were associated with ARDS in the cited works, we aimed to evaluate whether they were associated with a clinical phenotype similar to or associated with ARDS. Of the 735 GP associations initially found from catalog, we selected 140 GP associations from studies with 3000 or more individuals in both initial and replication cohort. The 140 selected GP pairs involved 32 candidate genes and 64 distinct traits (defined as having distinct identifier in Experimental Factor Ontology [https://www.ebi.ac.uk/ols/ontologies/efo]). The 64 traits were further simplified into 14 phenotypes. The list of gene–trait pair along with simplified phenotypes are shown in Supplementary file 2. The network of associations between genes and simplified phenotypes confirms that the candidate genes are mostly associated with key phenotypes of ARDS: cardiovascular disease, pulmonary function, and systemic inflammatory diseases (Figure 1). Genetic pleiotropy may exist between individuals with higher risk for ARDS and these clinical phenotypes. Conversely, pediatric patients recovered from ARDS require close monitoring of these clinical conditions prospectively.

### Summary of biomarkers by cell types at different phases

One of the most compelling reasons to characterize ARDS endotypes based on clinical factors, genetic risk, and biomarkers is to identify differences in pathobiology that may respond to different treatment strategies. For example, ANGPT2 is produced by activated endothelial cells, which destabilize the vascular junction in the setting of inflammation (Kim et al., 2016). Therefore, increased ANGPT2 has been proposed to have a causal role in ARDS due to endothelial activation (Reilly et al., 2018) and ANGPT or ANGPT2/1 levels associate with clinical outcome (Whitney et al., 2020a; Whitney et al., 2020b; Zinter et al., 2016).

**Figure 2.**
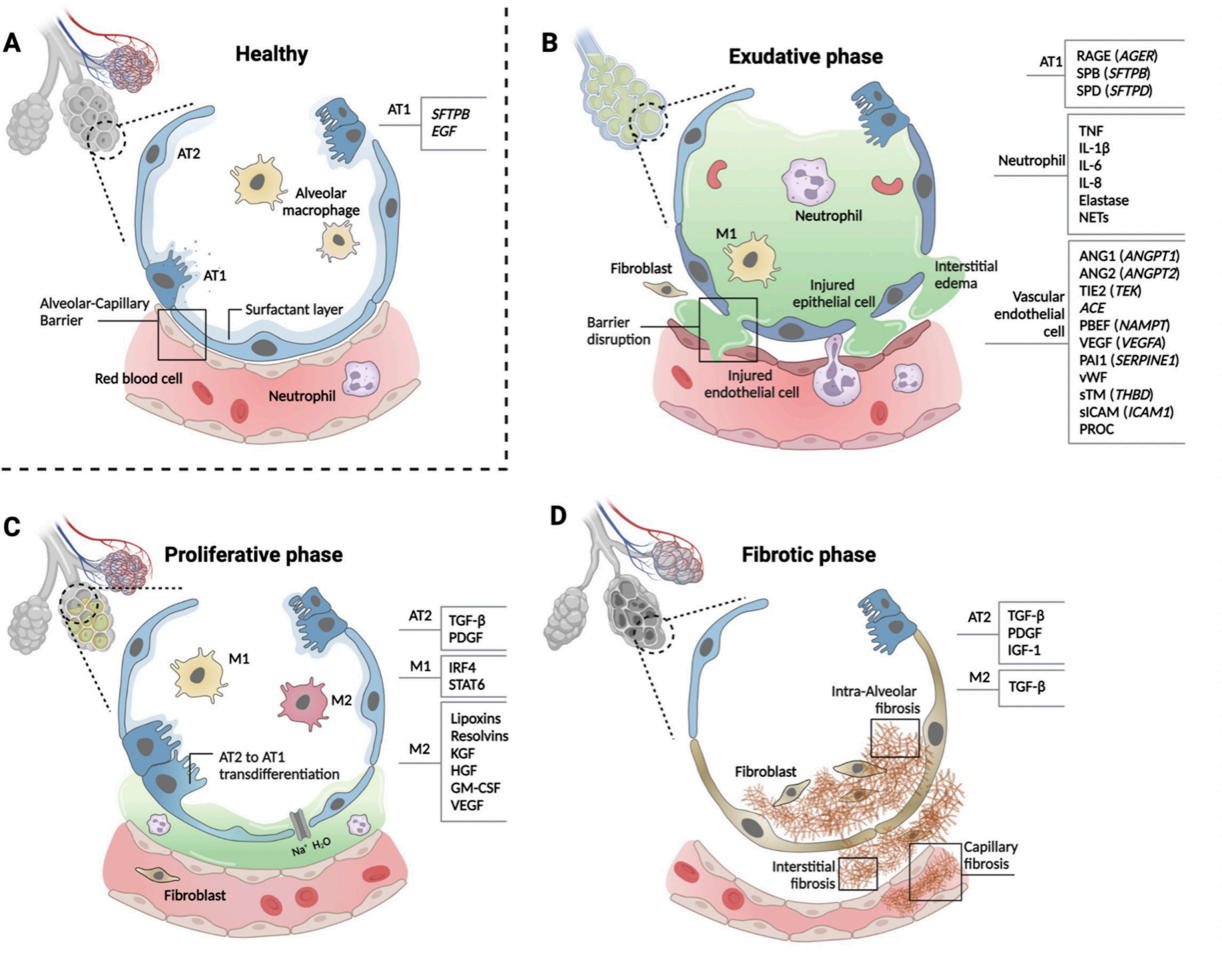
Phases of ARDS pathogenesis. Panel A shows alveolar–capillary interface in a state of good health. Panels B, C, and D, respectively, show the three phases of ARDS development with candidate biomarkers indicated by cell type. Candidate biomarkers indicate dysregulation following a physiologic insult with transition from (**A**) health to (**B**) exudative, (**C**) proliferative, and (**D**) fibrotic phases of illness. Patients may present to care in different phases of ARDS development, may progress through phases at different rates, and biology differ depending on cause of ARDS and patient age. Figure was created using Biorender.com. Abbreviations: ACE = angiotensin-converting enzyme; ANG1/ANGPT1 = angiopoietin 1; ANG2/ANGPT2 = angiopoietin 2; ARDS = acute respiratory distress syndrome; AT1 = type I alveolar cell; AT2 = type II alveolar cell; EGF = epidermal growth factor; GM-CSF = granulocyte-macrophage colony-stimulating factor; HGF = hepatocyte growth factor; IGF = insulin like growth factor; IL-1β = interleukin 1 beta; IL-6 = interleukin 6; IL-8 = interleukin 8; IRF4 = interferon regulatory factor 4; KGF = keratinocyte growth factor; M1 = type I macrophage; M2 = type II macrophage; NAMPT = nicotinamide phosphoribosyltransferase; NET = neutrophil extracellular trap; PAI1 = plasminogen activator inhibitor type 1; PBEF = pre-B-cell colony-enhancing factor; PDGF = platelet-derived growth factor; PROC = protein C, inactivator of coagulation factors Va and VIIIa; RAGE = receptor for advanced glycation end-products; SERPINE1 = serpin family E member 1; SFTPB = surfactant protein B; sICAM/ICAM1 = intercellular adhesion molecule 1; SPB = surfactant-associated protein B; SPD = surfactant-associated protein D; STAT6 = signal transducer and activator of transcription 6; sTM = soluble thrombomodulin; TGF-β = transforming growth factor beta; THBD = thrombomodulin; TIE2 (TEK) = tyrosine kinase with immunoglobulin- and EGF-like domains 1; TNF = tumor necrosis factor; VEGF/VEGFA = vascular endothelial growth factor A; vWF = von Willebrand Factor.

Specific putative biomarkers are more relevant to dysfunction of specific cell types at different phases of ARDS. Thus, we summarized ARDS biomarkers according to cell types implicated in the three major phases of ARDS progression (exudative, proliferative, and fibrotic; Mason et al., 2016 in Figure 2). When considering pathobiological evolution of ARDS through these proposed phases, it is important to note that (1) patients may present to care in different phases, (2) patients may progress through phases at different rates, and (3) scientific knowledge of ARDS phases is more developed for adults than children.

Alveolar cell types 1 and 2 (AT1 and 2) maintain the structure and function of alveoli (Figure 2, panel A), and multiple biomarkers indicate epithelial injury. Risk alleles for ARDS are reported in *SFTPB* and *EGF* genes that are important homeostatic function of AT1 cells. In the exudative phase, injured AT1 cells release soluble RAGE, SP-B, SP-D, CC-16 (*SCGB1A),* lamininγ2 (*LAMC2*), *KL-6 (MUC1*), and *KGF* (*FGF7*, Figure 2, panel B). In the proliferative phase, TGF-β and PDGF are released from AT2 cells (Figure 2, panel C). Activated AT2 cells continue to secrete TGF-β, PDGF, and IGF-1 to promote interstitial and intraalveolar fibrosis, which restrict lung capacity (Figure 2, panel D).

Systemic infection, inflammation, and direct lung injury activate vascular endothelial cells. Genetic susceptibility to ARDS has been reported for the genes that are highly expressed in endothelial cells and have functional significance for normal cellular function, including ANGPT2 (*ANGPT2*), TIE2 (*TEK*), *ACE*, PBEF (*NAMPT*), VEGF (*VEGFA*), and PAI1 (*SERPINE1*) (Acosta-Herrera et al., 2014). Protein biomarkers released from injured endothelial cells include ANGPT1, ANGPT2, VEGF, vWF, apolipoproteins, cell-free hemoglobin, PAI1, endothelial glycocalyx, sTM (*THBD*), sICAM (*ICAM1*), and PROC (Figure 2, panel B).

Tissue-resident macrophages defend against pathogens and pollutants entering alveoli. Alveolar macrophages are transformed to inflammatory M1-like macrophages by the activation of NF-κB signaling pathway then cytokines and chemokines, including TNF-α, IL-1β, IL-6, IL-8, CCL2, and CCL7, are released to recruit neutrophils in the exudative phase (Figure 2, panel B). Activated neutrophils secrete LTB4, ROS, MMPs, histones, and elastase and form NETs. In the proliferative phase, anti-inflammatory M2-like macrophages release lipoxins, resolvins, KGF, HGF, granulocyte-macrophage colony-stimulating factor (GM-CSF, and VEGF). AT2 cells proliferate and differentiate to AT1 cells by GM-CSF in this phase (Figure 2C). Regulatory T cells (Treg) also limit the inflammatory response. M2-like macrophages phagocytose apoptotic neutrophils (efferocytosis).

Genetic determinants of the innate immune response may be related to ARDS susceptibility; however, no risk alleles have been reproduced in two or more independent cohorts. Gene expression studies using peripheral blood monocytes (PBMCs) showed differential expression of *IL1R2*, *FTL*, *PI3*, and *S100A2* associated with an anti-inflammatory response. Protein biomarkers from immune cells, including IL-8, IL-6, IL-1β, IL-18, GM-CSF (*CSF2*), G-CSF (*CSF3*), sTNFR1 (*TNFRSR1A*), IL-10, and TNF (*TNF*), have been studies in ARDS; however, it is difficult to distinguish which cell types are responsible for these biomarkers. G-CSF is the dominant colony-stimulating factor released from diverse lung cells in response to proinflammatory cytokines that stimulates neutrophil development and differentiation. Proinflammatory M1-like macrophages release TNF, IL-6, IL-1β, and IL-23 while anti-inflammatory M2-like macrophages release TGFB1, IL-10, and IL-13.

In patients with ARDS, biomarkers have multiple actions across cell types affected at different stages of disease. Biomarker activity also varies according to the tissue type samples. Peripheral blood, tracheal aspirates, BAL fluid, lung biopsy, and autopsy samples have been used to discover biomarkers of ARDS; however, evaluation of abnormal biomarker levels should be interpreted in the context of cell types and disease stage. To this end, recent developments in maturation of single-cell profiling techniques such as scRNA-seq will be an important for further refinement of ARDS biomarkers.

### Cell type-specific expression of ARDS candidate genes in COVID-19

**Figure 3.**
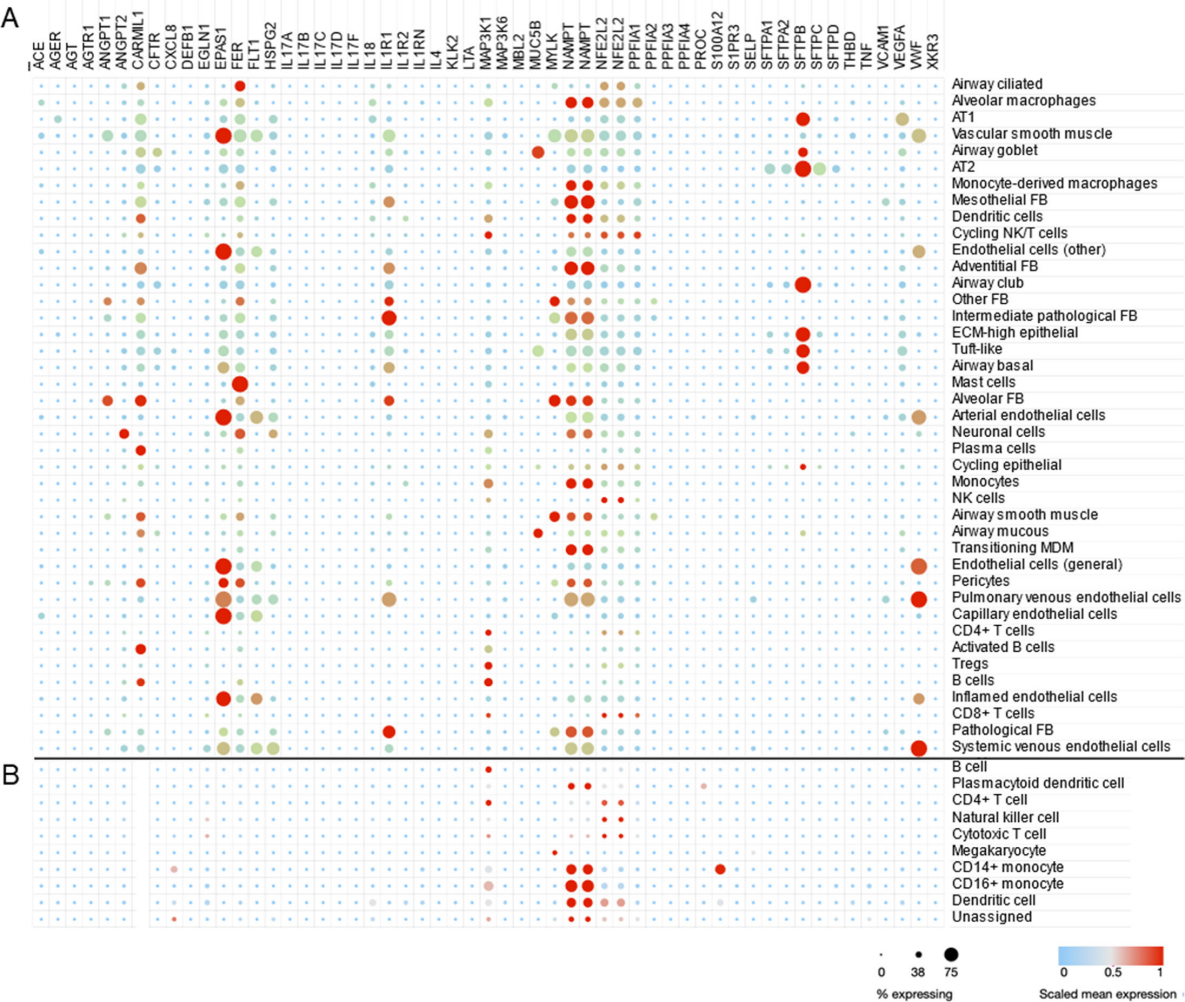
Cell type-specific expression pattern of ARDS candidate genes. Relative expression levels of ARDS candidate genes are shown across cell types from lung autopsy sample from ARDS caused by COVID-19 (**A**) and peripheral blood mononuclear cells a generally heathy individual (**B**). Expression levels are scaled to show relative levels across all cell types from 0 (blue) to 1 (red). The size of circle is proportional to the cells expressing a gene for each cell type (from 0% to 75%). AT1 and AT2: alveolar type I and II cells; FB: fibroblasts; ECM-high: high expression of extracellular matrix components (i.e., *COL6A3, COL1A2*, and *COL3A1*); MDM: monocyte-derived macrophages. Source data for this figure are provided in a table, Figure 3—source data 1. Figure 3—source data 1.Source data for Figure 3.

To demonstrate the utility of scRNA-seq and similar approaches, we use a dataset generated from adults with lethal ARDS secondary to COVID-19 (Melms et al., 2021). This dataset was composed of single-nucleus RNA sequencing of about 116,000 nuclei from lung autopsy samples that were highly inflamed with activated macrophages (Figure 3A). A total of 53 candidate genes were queried in the Single Cell portal at Broad Institute (available at https://singlecell.broadinstitute.org/single_cell/study/SCP1219/columbia-university-nyp-covid-19-lung-atlas, last accessed August 8, 2021) and scaled mean expression levels are shown for 41 detailed cell types defined by the original study. In Figure 3B, the same genes were highlighted in peripheral blood mononuclear cells (PBMCs) collected from healthy adults (Ding et al., 2020). The *CARMIL1* gene was highly expressed in diverse lung cells from severe COVID-19 patients; however, it was not expressed in PBMCs from healthy adults. The *EPAS1* gene (also known as *HIF2A*) was highly expressed in endothelial cells as well as AT1 and AT2 cells. The *FER* gene encodes a tyrosine kinase that is involved in the regulation of actin cytoskeleton. The expression of *FER* was observed in multiple cell types including AT1, AT2, alveolar macrophages, and mast cells in ARDS lung while peripheral immune cells are not actively express this gene. *IL1R1*—implicated in NET formation—was expressed in endothelial cells, epithelial cells, and fibroblasts. The highest expression of *VEGFA* was found in AT1 cells and *vWF* expression was high across endothelial and epithelial cells. Many candidate genes for ARDS were highly expressed in lung cells while few genes such as *MAP3K1* and *NAMPT* were also expressed in PBMC suggesting PBMC as a potential source of biomarker discovery (García-Laorden et al., 2017).

Recent studies using autopsy samples from adults with ARDS in the setting of COVID-19 infection uncovered key pathobiological changes in lung tissue. Lung tissue samples were found to be infiltrated with aberrantly activated monocyte-derived macrophages and alveolar macrophages that produced high levels of interleukin (IL)-1β and IL-6 (Xu et al., 2020; Jiang et al., 2020). Moreover, failure to transition from AT1 to AT2 cells impaired lung regeneration and repair (Melms et al., 2021). These discoveries have added important information to our understanding of ARDS pathobiology and could pave the way for the development of targeted therapies.

### Conclusion

**Figure 4.**
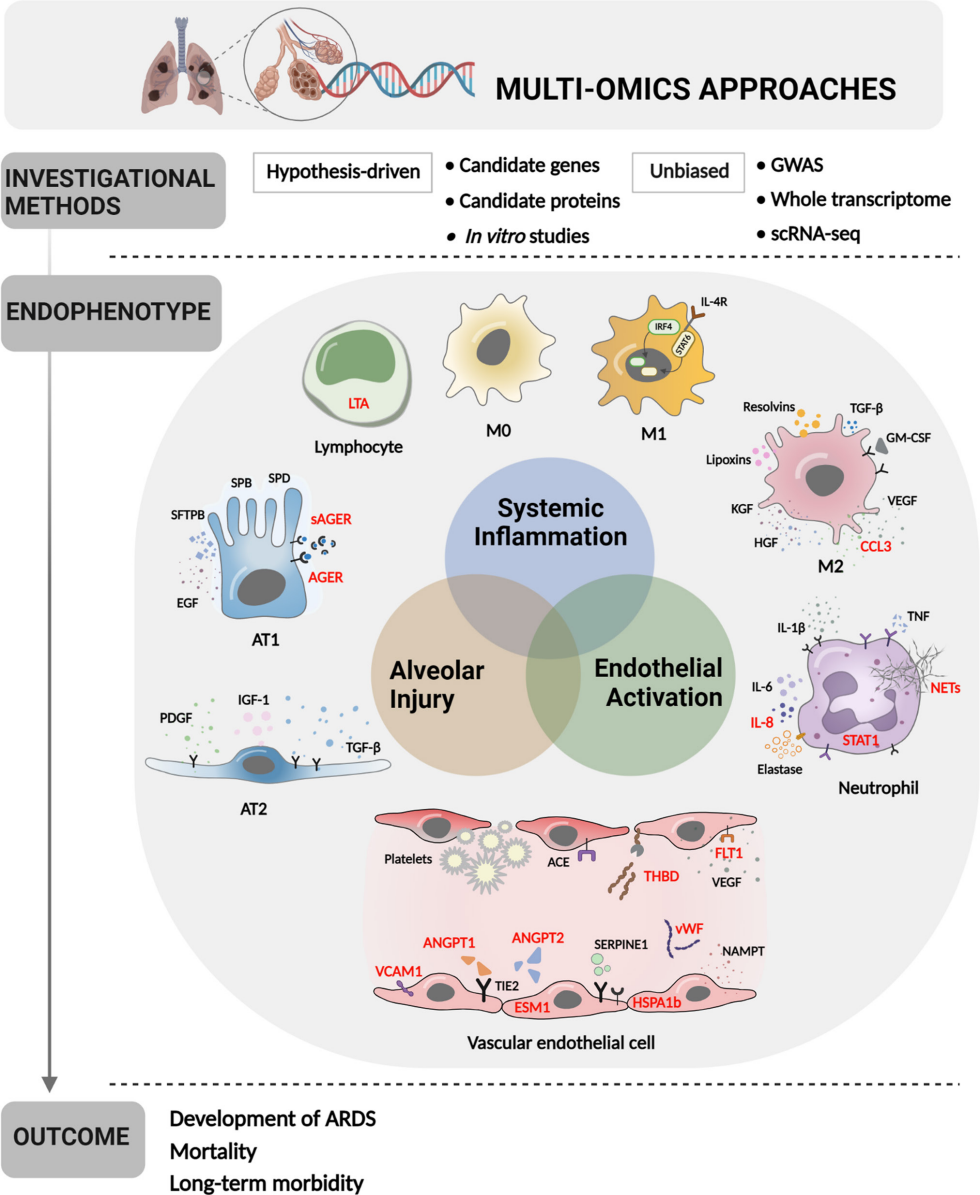
Multi-omics approaches to define endotypes of pediatric acute respiratory distress syndrome (PARDS). Investigational methods, including hypothesis-driven and unbiased investigations, have led to discovery of multiple contributions to ARDS pathobiology, including systemic inflammation, endothelial activation, and alveolar injury. These processes affect diverse cell and tissue types, including lymphocytes, macrophages, neutrophils, vascular endothelial cells, and two types of alveolar cells. The degree of systemic inflammation, endothelial activation, and alveolar injury across cell and tissue types has helped to define ARDS endotypes, which have been differentially associated with relevant clinical outcomes, including the development of ARDS in patients who have experienced clinical risk factors for the disease, mortality, and long-term morbidity from ARDS. Names of proteins implicated in PARDS as opposed to adult ARDS are depicted in red. Figure was created using Biorender.com. Abbreviations: ACE = angiotensin-converting enzyme; ANGPT1, -2 = angiopoietin 1, 2; CCL3 = C-C motif chemokine ligand 3; ESM1 = endothelial cell-specific molecule 1; FLT1 = fms-related receptor tyrosine kinase 1; GM-CSF = granulocyte-macrophage colony-stimulating factor; HGF = hepatocyte growth factor; HSPA1b = heat shock protein family A (Hsp70) member 1B; IL- = interleukin-; IRF4 = interferon regulatory factor 4; KGF = keratinocyte growth factor; LTA = lymphotoxin alpha; M0, M1, M2 = type 0, I, II macrophage; NAMPT = nicotinamide phosphoribosyltransferase; NET = neutrophil extracellular trap; SERPINE1 = serpin family E member 1; STAT1, -6 = signal transducer and activator of transcription-1,6; TGF-β = transforming growth factor beta; THBD = thrombomodulin; TIE2 = tyrosine kinase with immunoglobulin like and EGF like domains 1; VCAM1 = vascular cell adhesion molecule 1; VEGF = vascular endothelial growth factor A; vWF = von Willebrand Factor.

Prior studies have provided insight into the pathobiological pathways that determine whether ARDS develops in patients with risk factors and what clinical outcomes are experienced by adults and children with ARDS (Figure 4). Ability to define ARDS endotypes based on clinical and genomic markers may indicate heterogeneity in underlying pathobiology. However, prior literature is characterized by several limitations. First, much of the existing literature is based on relatively small cohorts, heterogeneous samples, and measurements confined to one or several timepoints during disease development. Adults are better represented than children in the current ARDS literature. Second, many studies were designed to test hypotheses based on prior knowledge, and therefore investigation of disease mechanism are not unbiased and could have missed alternative conclusions. Third, most studies evaluated peripheral blood with a few more recent investigations of lower airway cells and BAL fluid. However, not all genomic changes are reflected in circulating proteins and circulating protein changes, if present, may not reflect changes at the tissue level. Evaluation of lung tissue from autopsy specimens, while offering the most specificity investigations using scRNA-seq, is not always practical in PARDS, owing to its relatively low mortality rate. Lung biopsy of children with PARDS may confer unacceptable morbidity, so tracheal aspirate or BAL specimens may be most practical. PBMCs have been subjected to scRNA-seq in several adult ARDS studies that demonstrated feasibility; however, differences between the blood and lung compartments deserve further study. A logical next step to address some of these issues is scRNA-seq evaluation of tracheal aspirate samples as well as peripheral blood from a well-characterized cohort of children with PARDS, who can be longitudinally sampled and followed. As technological advances continue, new methods should continue to be applied in order to elucidate the pathobiology of PARDS as it develops and resolves so that disease-targeted therapies can be developed.

### Ideas and speculation: opportunities for new discoveries with scRNA-seq

PARDS is caused by diverse exogenous pathogens and stressors, though genetic susceptibility may play a role in which at-risk patients develop the disease. Although risk alleles in candidate genes from a limited number of studies with small sample sizes have been reported for PARDS, most studies were underpowered. Phenotypes associated with candidate genes are diverse across multiple organ systems including heart, lung, kidney, and immune systems. scRNA-seq and other unbiased characterization methods at cell level provide great potential to identify cell types and markers in genes across multiple affected tissues and circulating immune cells.

## Additional files

Supplementary file 1. Key adult studies.

Supplementary file 2. Phenotypes associated with ARDS candidate genes from genome-wide association studies including at least 3000 samples.
