# Supplementary material for: Evolution of multiple omics approaches to define pathophysiology of pediatric acute respiratory distress syndrome
Source: eLife. 2022 Aug 1;11:e77405. doi: 10.7554/eLife.77405 (PMC9342956; doi:10.7554/eLife.77405)
Supplement: Supplementary file 1. [file elife-77405-supp1.docx]

Supplementary File 1: Key Adult Studies

| Approach | Candidate Gene(s) | Sample Size | Findings (Adjusted OR and CI) | Author, Journal*, Year |
| --- | --- | --- | --- | --- |
| HYPOTHESIS-DRIVEN |  |  |  |  |
| Candidate Gene | *ACE* | 84 German adults with ARDS, 200 controls (total 284) | DD genotype associated with increased mortality compared to II: hazard risk 5,7, CI 1.7-19.2 | Adamzik et al., Eur Respir J, 2007 |
|  | many | 2 cohorts: 225 Spanish adults with sepsis-associated ARDS, 899 healthy controls; 661 adults of Western European descent with sepsis-associated ARDS, 234 controls (total 2019) | *FLT1* SNP rs9513106 associated with reduced susceptibility to ARDS, AOR for both cohorts 0.8, CI 0.7-0.9 | Hernandez-Pacheco et al., Intensive Care Med Exp, 2018 |
|  | *ACE* | 4 cohorts Caucasian: 96 ARDS, 88 non-ARDS intubated respiratory failure, 174 after coronary artery bypass graft, 1906 healthy controls (total 2264) | DD genotype and D allele more frequent in ARDS than other groups, D allele associated with increased mortality in ARDS | Marshall et al., Am J Respir Crit Care Med, 2002 |
|  | *ANGPT2* | 2 cohorts: 222 African American adults with trauma without ALI, 600 European American adults with trauma with or without ALI | Stage 1: Five SNPs including *ANGPT2* SNP rs1868554 associated with ALI, OR 2.6, CI 1.7-4.1. Stage 2: rs1868554 was replicated with OR 1.2, CI 1.1-1.4 | Meyer et al., Am J Respir Crit Care Med, 2011 |
|  | *IL1R1* | 3041 with ARDS risk factors | *IL1R1* SNP rs315952C associated with reduced susceptibility to ARDS, AOR 0.8, CI 0.7-0.9 | Meyer et al., Am J Respir Crit Care Med, 2013 |
|  | *NAMPT* | 126 with SIRS ARDS, 126 healthy controls (total 252) | *NAMPT* SNP rs61330082 associated with reduced susceptibility to ARDS, AOR 0.6, CI 0.4-1.0 | O’Mahony et al., PLoS One, 2012 |
|  | *ABO* | 732 with trauma, 976 with severe sepsis (total 1708) | AA genotype associated with ARDS in White but not Black patients: AOR 1.9, CI 1.1-3.1 | Reilly et al., Chest, 2014 |
|  | *ANGPT2* | 2 cohorts: 403 and 254 with sepsis, some with ARDS (total 657) | Two *ANGPT2* SNPs associated with ARDS in European ancestry patients: rs2442608 AOR 1.4, CI 1.0-1.9, rs2442630 AOR 5.5, CI 1.7-17.8 | Reilly et al., Intensive Care Med, 2018 |
|  | *ABO* | 2 trauma cohorts (1212, 1471) and 1 sepsis (1027) cohort, some with ARDS (total 3701) | A1 compared to O haplotype associated with ARDS: trauma cohort 1 AOR 1.6, CI 1.1-2.3, trauma cohort 2 AOR 1.4, CI 1.0-1.9, sepsis cohort AOR 1.5, CI 1.0-2.3 | Reilly et al., JCI, 2021 |
|  | *PROC, PROCR, THBD* | 320 with ARDS | Increased mortality associated with two *PROC* and one *PROCR* SNPs: rs1042580 AOR 2.8, 1.2-6.1, rs3716123 AOR 6.3, CI 1.6-24.8, rs9574 AOR 2.8, CI 1.1-7.3 | Sapru et al., Crit Care 2016 |
|  | *ANGPT2* | 1529 with ARDS risk factors | ARDS associated with *ANGPT2* SNP rs2515475 AOR 1.3, CI 1.0-1.6, especially indirect ARDS AOR 1.8, CI 1.2-2.6; ARDS associated with haplotype TT rs2515475-T and rs2959811-T AOR 1.4, CI 1.1-1.9, especially indirect ARDS AOR 1.9, CI 1.2-2.9. | Su et al., Intensive Care Med, 2009 |
|  | *POPDC3, FAAH* | 1717 acute lung injury, 765 trauma, 838 pneumonia sepsis (total 3320) | Decreased ARDS in direct lung injury associated with *POPDC3* SNP rs1190286 AOR 0.6; increased ARDS in trauma-associated indirect lung injury associated with FAAH SNP rs324420, AOR 1.7 in meta-analyses. | Tejera et al., J Med Genetics, 2012 |
| Candidate Protein | *IL8, GCSF, GMCSF* | 31 ARDS, 21 healthy controls | IL8, GCSF, GMCSF increased in BAL fluid from ARDS patients, GCSF higher in nonsurvivors | Aggarwal et al., Eur Respir J, 2000 |
|  | many | 218 uninflamed and 236 reactive patients with ARDS (total 454) | High IL6, IFNG, ANGPT1, ANGPT2, SERPINE1 defined reactive phenotype, associated with higher mortality, organ failure, and indirect lung injury | Bos et al., Thorax, 2017 |
|  | *ANGPT2, vWF* | 931 ALI | ANGPT2 associated with mortality and modified by presence of infection (Infected: OR 2.4, CI 1.6-3.8 per log increase; non-infected: OR 2.3, 1.5-3.4 if ANGPT2 increased from day 0 to 3) | Calfee et al., Crit Care Med, 2012 |
|  | many (protein and clinical markers) | 2 cohorts: 473, 549 with ARDS (total 1002) | Increased IL6, IL8, TNFRSF1A, SERPINE1 and clinical markers identified hyperinflammatory subphenotype associated with higher mortality, fewer ventilator-free and organ failure-free days. Differential effect of PEEP on mortality by subphenotype | Calfee et al., Lancet Respir Med, 2014 |
|  | *ANGPT2, AGER, SFTPD, IL6, IL8* | 2 cohorts: 100 with sepsis ARDS, 853 with ARDS (total 953) | High SFTPD (Cohort 1: AOR 2.46, CI 1.3-4.5, Cohort 2: AOR 1.32, CI 1.2-1.5), low ANGPT2 (1: AOR 0.5, CI 0.3-1.0, 2: AOR 0.6, CI 0.5-0.7) associated with ARDS with direct lung injury in both cohorts | Calfee et al., Chest, 2015 |
|  | many (protein and clinical markers) | 1000 ARDS | Higher IL6, IL8, TNFRSF1A, ANGPT2, AGER and clinical markers identified subphenotype with higher mortality, fewer ventilator-free and organ failure-free days, which had lower mortality with liberal fluid administration | Famous et al., Am J Respir Crit Care Med, 2016 |
|  | *AGER* | 2 sepsis cohorts, some with ARDS: 558, 843 (total 1401) | Plasma AGER had a causal effect on ARDS risk (β 0.5, CI 0.1-0.9 per log increase) | Jones et al., Am J Respir Crit Care Med, 2020 |
|  | many (protein and clinical markers) | 745 ARDS | TNFRSF1A, IL8, bicarbonate identified  hyperinflammatory subphenotype associated with increased 60- and 90-day mortality with no impact of rosuvastatin on outcome | Sinha et al., Intensive Care Med, 2018 |
|  | many (protein and clinical markers) | 4 cohorts, 2737 total | PROC, IL8, bicarbonate identified hyperinflammatory ARDS phenotype with distinct clinical features and outcomes, including differential survival with simvastatin in one cohort | Sinha et al., Lancet Respir Med, 2020 |
|  | *AGER* | 3 cohorts: 11 hydrostatic pulmonary edema, 22 ALI/ARDS, 11 healthy controls (total 44) | AGER highest in patients with ALI/ARDS compared to hydrostatic pulmonary edema or healthy controls | Uchida et al., Am J Respir Crit Care Med, 2006 |
|  | many | 2 cohorts of patients with sepsis: 100 with ARDS, 100 without ARDS (total 200) | ARDS predicted by SFTPD, AGER, IL7, SCGB1A1, IL6 (AUC 0.75, CI 0.7-0.8) | Ware et al., Crit Care, 2013 |
| *In Vitro* Studies |  | Neutrophils and BAL fluid from 25 ARDS and 21 healthy controls | ARDS neutrophils with lower apoptosis, higher NET formation. Increased NETs in neutrophils exposed to ARDS but not control BAL fluid. Macrophage activity decreased in ARDS, but enhanced with metformin or HMGB1 neutralization | Grégoire et al., Eur Respir J, 2018 |
|  |  |  |  |  |
| UNBIASED |  |  |  |  |
| Genome-Wide Association |  | 232 African American patients with ARDS, 162 at-risk controls | *SELPLP* SNP rs2228315 associated with ARDS: OR 1.9, CI 1.3-2.7 | Bime et al., Am J Respir Crit Care Med, 2018 |
|  |  | 2 GWA Phases: 600 European-American adults with trauma-associated ALI and 2266 European-American controls; 212 trauma-associated ALI, 238 at-risk trauma non-ALI controls (total 3316) | *PPFIA1* SNP rs47191 associated with ALI due to trauma, AOR Phase I 1.2, p=0.01; AOR Phase 2 1.5, p=0.0045 | Christie et al., PLoS One, 2012 |
|  |  | 1935 European adults with sepsis, some with ARDS | *FLT1* SNP rs9508032 associated with reduced susceptibility to ARDS: OR 0.6, CI 0.4-0.9 | Guillen-Guio et al., Lancet Resp Med, 2020 |
| Gene Expression Profiling |  | 210 with sepsis ARDS: 128 inflamed, 28 uninflamed | DEGS in inflamed phenotype associated with upregulation of oxidative phosphorylation pathways reflecting mitochondrial dysfunction, uninflamed phenotype with upregulation of mitogen-activated protein kinase pathways | Bos et al., Am J Respir Crit Care Med, 2019 |
|  |  | 88 with ARDS | Transcripts related to inflammosome were increased | Dolinay et al., Am J Respir Crit Care Med, 2012 |
|  |  | 11 with ARDS (5 status post HCST) | DEGs in HSCT with ARDS associated with response to type I interferon | Englert et al., Respir Res, 2019 |
|  |  | 57 with sepsis: 29 with ARDS, 28 without | DEGs in sepsis ARDS associated with initial neutrophil response to infection | Kangelaris et al., Am J Physiol Lung Cell Mol Physiol, 2015 |
|  |  | Kangelaris cohort (above) | DEGs in sepsis ARDS associated with mitotic cell cycle changes | Wang et al., Biol Res, 2016 |
| Metabolomics |  | Pulmonary edema fluid from 16 ARDS and 13 control | High metabolite endotype associated with high mortality from ARDS | Rogers et al., Am J Respir Crit Care Med, 2017 |

Abbreviations: ACE = angiotensin-converting enzyme; AGER = advanced glycosylation end-product specific receptor; ALI = acute lung injury; ANGPT2 = angiopoietin 2; AOR = adjusted odds ratio; AUC = area under the receiver operator characteristic curve; ARDS = acute respiratory distress syndrome; BAL = bronchoalveolar lavage; CI = 95% confidence interval; DEG = differentially-expressed genes; FAAH = fatty acid amide hydrolase; FLT1 = fms related receptor tyrosine kinase 1; GCSF = granulocyte colony stimulating factor; GMCSF = granulocyte macrophage colony stimulating factor; HCST = hematopoietic stem cell transplant; HMGB1 = high mobility group box 1; ICU = intensive care unit; IFNG = interferon gamma; IL = interleukin; IL1R1 = interleukin 1 receptor type 1; NAMPT = nicotinamide phosphoribosyltransferase; NET = neutrophil extracellular trap; OR = unadjusted odds ratio; POPDC3 = popeye domain containing 3; PROC = protein C; PROCR = protein C receptor; PEEP = positive end expiratory pressure; PPFAIA1 = protein tyrosine phosphatase, receptor type F polypeptide interacting protein A1; SCGB1A1 = secretoglobin family 1A member 1; SELPLG = selectin P ligand; SERPINE1 = serpin family E member 1; SFTP = surfactant protein; SIRS = systemic inflammatory response syndrome; SNP = single nucleotide polymorphism; THBD = thrombomodulin; TNFRSF1A = TNF receptor superfamily member 1A; VWF = von Willebrand Factor

*Journal Titles are abbreviated according to U.S. National Library of Medicine convention.
