# Supplementary material for: Evolution of multiple omics approaches to define pathophysiology of pediatric acute respiratory distress syndrome
Source: eLife. 2022 Aug 1;11:e77405. doi: 10.7554/eLife.77405 (PMC9342956; doi:10.7554/eLife.77405)
Supplement: Supplementary file 2. [file elife-77405-supp2.docx]

**Supplementary File 2: Phenotypes associated with ARDS candidate genes from genome-wide association studies including at least 3,000 samples**

| **Gene symbol** | **Merged phenotypes linked to the gene [phenotypes as listed in GWAS catalog]** |
| --- | --- |
| ACE | Cardiovascular Health/Disease [diastolic blood pressure; systolic blood pressure] |
| AGER | Pulmonary Function/Disease [vital capacity; forced expiratory volume; FEV/FEC ratio; chronic obstructive pulmonary disease; pulmonary  function measurement; peak expiratory flow] |
| AGT | Cardiovascular Health/Disease [coronary artery disease; systolic blood pressure; diastolic blood pressure]  Health-Related Behavior [smoking status measurement; alcohol drinking] |
| ANGPT1 | Anthropometric Measurement [body mass index]  Neurologic Injury [white matter hyperintensity measurement]  Ophthalmologic Disease [open-angle glaucoma; intraocular pressure measurement] |
| ANGPT2 | Renal Function [glomerular filtration rate] |
| CARMIL1 | Cardiovascular Health/Disease [coronary artery disease]  Hematologic Measurement [platelet count]  Mental Health [neuroticism measurement; depressive symptom measurement; wellbeing measurement]  Systemic Inflammation [mucocutaneous lymph node syndrome] |
| CFTR | Anthropometric Measurement [waist circumference]  Cardiovascular Health/Disease [coronary artery disease] |
| CXCL8 | Systemic Inflammation [interleukin-8 measurement] |
| EPAS1 | Oncologic Disease [renal cell carcinoma] |
| FER | Anthropometric Measurement [BMI-adjusted waist circumference; pulse pressure measurement; systolic blood pressure; body height]  Health-Related Behavior [smoking behavior] |
| FLT1 | Cardiovascular Health/Disease [coronary artery disease]  Vascular Health [placenta growth factor measurement] |
| HSPG2 | Anthropometric Measurement [body height] |
| IL17A | Anthropometric Measurement [body height] |
| IL18 | Systemic Inflammation [interleukin 18 measurement] |
| IL1R1 | Allergic Dermatitis [atopic eczema]  Cardiovascular Health/Disease [ST2 protein measurement]  Hematologic Measurement [fibrinogen measurement]  Liver Function Test [serum alanine aminotransferase measurement]  Pulmonary Function/Disease [asthma; childhood onset asthma] |
| IL4 | Pulmonary Function/Disease [asthma] |
| KLK2 | Oncologic Disease [prostate specific antigen measurement; prostate carcinoma] |
| LTA | Anthropometric Measurement [pulse pressure measurement]  Pulmonary Function/Disease [asthma]  Systemic Inflammation [oral ulcer; mucocutaneous lymph node syndrome; Crohn's disease] |
| MAP3K1 | Oncologic Disease [breast carcinoma] |
| MBL2 | Ophthalmologic Disease [age-related macular degeneration] |
| NFE2L2 | Renal Function [glomerular filtration rate] |
| PPFIA1 | Cardiovascular Health/Disease [coronary artery disease] |
| PPFIA2 | Anthropometric Measurement [lean body mass]  Cardiovascular Health/Disease [resting heart rate] |
| PPFIA4 | Cardiovascular Health/Disease [atrial fibrillation] |
| SELP | Ophthalmologic Disease [optic disc size measurement]  Vascular Health [blood protein measurement] |
| SFTPA2 | Pulmonary Function/Disease [FEV/FEC ratio] |
| SFTPD | Pulmonary Function/Disease [FEV/FEC ratio; chronic obstructive pulmonary disease]  Systemic Inflammation [rheumatoid arthritis] |
| THBD | Hematologic Measurement [thrombomodulin measurement] |
| TNF | Anthropometric Measurement [pulse pressure measurement]  Systemic Inflammation [Crohn's disease] |
| VCAM1 | Systemic Inflammation [multiple sclerosis] |
| VEGFA | Anthropometric Measurement [BMI-adjusted waist-hip ratio; BMI-adjusted waist circumference; body mass index; waist-hip ratio;  anthropometric measurement]  Cardiovascular Health/Disease [triglyceride measurement; high density lipoprotein cholesterol measurement; adiponectin measurement;  coronary artery disease]  Health-Related Behavior [physical activity measurement; smoking behavior; physical activity; sleep duration; alcohol drinking; alcohol  consumption measurement]  Hepatic Function Test [aspartate aminotransferase measurement; serum alanine aminotransferase measurement]  Oncologic Disease [prostate specific antigen measurement]  Renal Function [hyperuricemia] |
| VWF | Hematologic Measurement [factor VIII measurement; von Willebrand factor measurement] |
